# Author Correction: Homologous Pairs of Low and High Temperature Originating Proteins Spanning the Known Prokaryotic Universe

**DOI:** 10.1038/s41597-023-02685-z

**Published:** 2023-10-31

**Authors:** Evan Komp, Humood N. Alanzi, Ryan Francis, Chau Vuong, Logan Roberts, Amin Mosallanejad, David A. C. Beck

**Affiliations:** 1 Department of Chemical Engineering, University of Washington, Seattle, USA; 2 Department of Biochemistry, University of Washington, Seattle, USA; 3 eScience Institute, University of Washington, Seattle, USA; 4 Paul G. Allen School of Computer Science, University of Washington, Seattle, USA

**Keywords:** Proteins, Bioinformatics, Protein design, Software

Correction to: *Scientific Data* 10.1038/s41597-023-02553-w, published online 07 October 2023

In this article the author name Humood N. Alanzi was incorrectly written as Humood H. Alanzi. In addition, the author name Amin Mosallanejad was incorrectly written as Amin Mossallanejad. The original article has been corrected.

